# Mechanisms of Immunothrombosis by SARS-CoV-2

**DOI:** 10.3390/biom11111550

**Published:** 2021-10-20

**Authors:** María Teresa Hernández-Huerta, Alma Dolores Pérez-Santiago, Laura Pérez-Campos Mayoral, Luis Manuel Sánchez Navarro, Francisco Javier Rodal Canales, Abraham Majluf-Cruz, Carlos Alberto Matias-Cervantes, Eduardo Pérez-Campos Mayoral, Carlos Romero Díaz, Gabriel Mayoral-Andrade, Margarito Martínez Cruz, Judith Luna Ángel, Eduardo Pérez-Campos

**Affiliations:** 1 CONACyT, Facultad de Medicina y Cirugía, Universidad Autónoma “Benito Juárez” de Oaxaca, Oaxaca 68020, Mexico; marte-hh28@hotmail.com (M.T.H.-H.); carloscervantes.ox@outlook.com (C.A.M.-C.); 2 Grupo de Investigación Biomedicina y Salud, Facultad de Medicina y Cirugía, Universidad Autónoma “Benito Juárez” de Oaxaca, Oaxaca 68020, Mexico; epcm@live.com.mx (E.P.-C.M.); carlos.rom.74he@gmail.com (C.R.D.); drmayoral@gmail.com (G.M.-A.); 3 Tecnológico Nacional de México/IT Oaxaca, Oaxaca de Juárez, Oaxaca 68030, Mexico; aperez_santiago@hotmail.com (A.D.P.-S.); martinezcu9@hotmail.com (M.M.C.); 4 Centro de Investigación Facultad de Medicina UNAM-UABJO, Facultad de Medicina y Cirugía, Universidad Autónoma “Benito Juárez” de Oaxaca, Oaxaca 68020, Mexico; rrodal22@gmail.com; 5 Facultad de Medicina y Cirugía, Universidad Autónoma “Benito Juárez” de Oaxaca, Oaxaca 68020, Mexico; sluismanuel81@hotmail.com; 6 Instituto Mexicano del Seguro Social, Ciudad de Mexico 06600, Mexico; amajlufc@gmail.com; 7 Hospital General Dr. Aurelio Valdivieso, Oaxaca de Juárez, Oaxaca 68000, Mexico; yuricoch@hotmail.com; 8 Laboratorio de Patología Clinica “Eduardo Pérez Ortega”, Oaxaca de Juárez, Oaxaca 68000, Mexico

**Keywords:** pathogen-associated molecular patterns, damage-associated molecular patterns, extracellular DNA, extracellular RNA, SARS-CoV-2, immunothrombosis

## Abstract

SARS-CoV-2 contains certain molecules that are related to the presence of immunothrombosis. Here, we review the pathogen and damage-associated molecular patterns. We also study the imbalance of different molecules participating in immunothrombosis, such as tissue factor, factors of the contact system, histones, and the role of cells, such as endothelial cells, platelets, and neutrophil extracellular traps. Regarding the pathogenetic mechanism, we discuss clinical trials, case-control studies, comparative and translational studies, and observational studies of regulatory or inhibitory molecules, more specifically, extracellular DNA and RNA, histones, sensors for RNA and DNA, as well as heparin and heparinoids. Overall, it appears that a network of cells and molecules identified in this axis is simultaneously but differentially affecting patients at different stages of COVID-19, and this is characterized by endothelial damage, microthrombosis, and inflammation.

## 1. Introduction

In December 2019, the infectious outbreak of a new human coronavirus (SARS-CoV-2) responsible for acute respiratory syndrome was detected in Wuhan, China [1]. The WHO subsequently identified it as “the new novel coronavirus 2019”, or COVID-19 [2]. According to information available, SARS-CoV-2 has caused more than 235 million cases worldwide and more than 4 million deaths as of 5 October 2021 (https://coronavirus.jhu.edu/map.html) (accessed on 9 October 2021) [3]. Moreover, the scenario for 1 January 2022 is expected to exceed 5.8 million COVID-19 deaths globally [4].

COVID-19 shows heterogeneous clinical expression, which is associated with thrombosis and microangiopathy. There are various opposing theories related to the virus. On the one hand, it could be associated with intravascular coagulation [5]. On the other hand, it could correspond to complement-mediated thrombotic microangiopathies [6,7]. Hypercoagulation [8], platelet hyperactivity [9], and abnormal fibrinolysis [10,11] might explain the diversity of macrovascular and microvascular thrombosis expression, depending on the study method. In the selection criteria for study subjects and the general population, the frequency of thrombosis is variable. For example, results from autopsies report that in 87% of microthrombosis cases [12], coagulopathy is found in up to 50% of fatalities [13], and macrothrombosis, such as deep vein thrombosis and pulmonary thromboembolism, is found in up to 40% [14,15].

**Figure 1:**
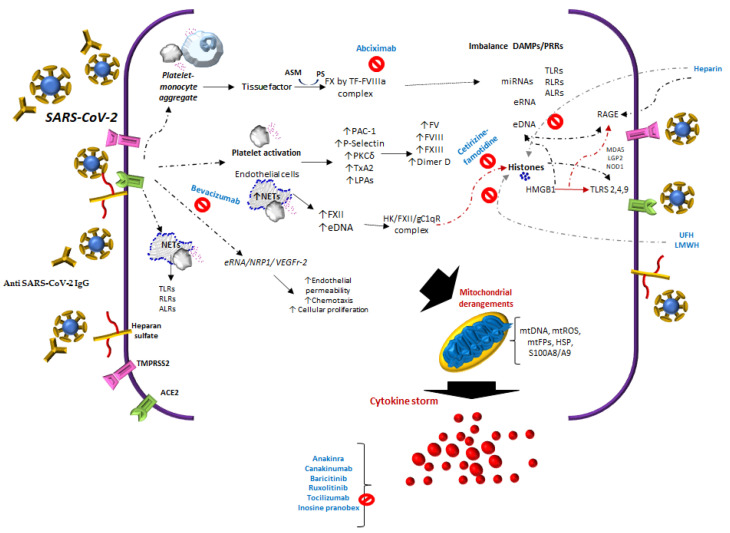
The reported studies of COVID-19 infer that there are multiple activation or inhibition routes in platelets and endothelium, this involves the release of tissue thromboplastin (TF), with the participation of monocyte-platelet, neutrophil-platelet aggregates, complement-TF-NETs, and histones. In addition, to immune complexes SARS-CoV-2 spike/anti-spike IgG, anti-PF4/heparin IgG antibodies and antiphospholipid antibodies.

Several signaling routes have been reported to play a role in the mechanisms of immunothrombosis or thrombo-inflammation [16,17] and cytokine storms in COVID-19 (Figure 1). The sequence of these events in SARS-CoV-2 infection is related to the interactions of different cells and molecules (Table 1 and Table 2), such as Angiotensin-Converting Enzyme 2 (ACE2), tissue thromboplastin or tissue factor (TF), neutrophil extracellular traps (NETs), extracellular DNA (eDNA) and RNA, histones [18], anti-PF4/heparin IgG antibodies, antiphospholipid antibodies, neutrophil-platelet aggregates, and monocyte-platelet, among others.

### 1.1. Factors of the Contact System

Recently, some molecules of the contact system (prekallikrein (PK), high-molecular-weight kininogen (HK), and factor XII (FXII)) have been reported to form an HK/FXII/gC1qR complex [19,20] which could participate in coagulation or inflammation. The gC1qR is a highly anionic multifunctional protein that participates in different mechanisms, including inflammation and vascular injury [21].

In COVID-19, specifically, the expression of FXIIa increases in lung tissue. In addition, this factor is colocalized with NETs in the lungs, indicating that the accumulation of NETs leads to greater activation of FXII due to a defect in the clearance of NETs by DNases, contributing to procoagulant activity [22]. This is also related to the activity of FXII in the blood coagulation system and increases in DNA and H4 histones [23]. Histones contribute to microvascular thrombosis and competitively inhibit plasmin to delay fibrinolysis [24].

Effects of factors of the contact system may also explain how coagulation, the kallikrein-kinin system, and inflammation molecules participate together as defense mechanisms favoring procoagulant mechanisms [25].

Clinical trials of this pathway of complement and kallikrein-kinin system activation use human recombinant C1 esterase inhibitor [26].

### 1.2. Tissue Factor

Factor III is a membrane protein that acts in the extrinsic pathway of coagulation, forming the FVIIa/TF complex [27]. The expression of tissue thromboplastin (also known as coagulation factor III), is a complex mechanism, a de-encryption of TF, which includes phospholipid scramblase and acid sphingomyelinase, i.e., the process transfers phosphatidylserine to the outer plasma membrane in monocytes for the efficient activation of FX by the TF-FVIIa complex [28]. Increases in TF release results in hypercoagulability and venous and arterial thromboembolism. TF is known to be released from different sources such as the alternative polarization macrophages [29] and microvesicles from endothelial cells [30]. The release of TF results from the formation of platelet-monocyte aggregates observed in severe COVID-19 [31]. Moreover, activated platelets induce NETs that carry FT [18]. In COVID-19, increased circulating extracellular vesicle TF activity has been reported, which correlates with the markers of thrombosis such as D-dimer [32]. It is important to point out that TF expression may be inhibited by platelet P-selectin (CD62P) neutralization or integrin αIIb/β3 blocking, as with abciximab [31].

### 1.3. Neutrophil Extracellular Traps and Molecule Release

Circulating neutrophils infected with SARS-CoV-2 release elevated levels of neutrophil-derived extracellular traps in the blood, trachea, and the lungs [33]. They also deliver all the contents of the nucleosomes, i.e., H2A, H2B, H3, and H4 histones, DNA [34], extracellular circulating viral micro-RNAs [35,36], and TF [25]. In COVID-19, the platelet/NETs/TF/thrombin axis is enhanced by complement activation [33]. Not only do neutrophils release eDNA, but also macrophages, eosinophils, and mast cells. These appear at different stages of thrombosis [37,38], and similarly in tumor cells [34].

In COVID-19 patients, platelet activation products, such as TXB2 and proteins from platelet α-granules PF4/CXCL4 and PDGF, are also released and found in tracheal aspirates [31]. Furthermore, it has been found that NETs can serve as a platform for the activation of contact factors of the intrinsic coagulation pathway [22], such as FXII, FXI, and PK, in the lung parenchyma.

Of the molecules with NETs that are released, it has been suggested that free DNA may be the cause of a more severe pathology in COVID-19 [39]. Moreover, the manifestations of severity could be related not only to eDNA, but also to other alarmins, such as extracellular heat-shock proteins and HMGB1, mentioned above, in addition to diverse self-nucleic acids, including nuclear DNA, ribosomal RNA, extracellular RNA (eRNA), micro-RNAs, and histones [40], Figure 1.

NETs are cytotoxic and procoagulant, in part due to the release of DNA-histone complexes and double-stranded DNA, histones, and HMGB1 [41,42]. It is understood that the increase in DNA-binding proteins, extracellular ribosomal RNA, and micro-RNAs, may be related to thrombosis [43,44].

In COVID-19, eDNA from NETs and histones could also explain thrombosis in severe forms [45]. The half-life of eDNA is around 4–30 min [46]. Its clearance is regulated by different factors, such as 1) Serine proteases, e.g., Factor VII activating protease; cysteine proteases, e.g., caspase-activated DNAse [47]; DNASE1; and deoxyribonuclease 1-like 3 (DNASE1L3) [48]. A deficiency in any of these nucleases causes the inability to remove blood clots [49]. 2) Receptors, such as those for advanced glycation end-products. In addition, Toll-like receptors 7 and 9 are sensors for eDNA in plasmacytoid dendritic cells (PDC) which have a huge capacity for producing type I (IFN-α) and type III (IFN-λ) interferons; 100–1000 more than other cells [50]. Type III IFN shows greater activity against SARS-CoV-2 [51]. PDC and NK cells in the presence of interferon inducers, such as RNA-containing immune complexes, produce tumor necrosis factor -α (TNF-α) and IL-6, among other pro-inflammatory cytokines, as in systemic lupus erythematosus. These cytokines are inhibited by interleukin -1 receptor-associated kinase 4 small molecule inhibitor, and also by hydroxychloroquine [52]. In addition, eRNA interacting with high affinity with vascular endothelial growth factor (VEGF) leads to the activation of VEGF-receptor 2/neuropilin-1 complex [42]. This complex increases endothelial permeability, chemotaxis, and cellular proliferation [53]. Moreover, some VEGF isoforms, such as the VEGF165 isoform, stimulate vascular growth and produce hyperpermeability. In order to block hyperpermeability in patients with severe COVID-19, clinical trials have been generated using bevacizumab as an anti-vascular endothelial growth factor [54,55].

A crucial point of NETs is the equilibrium or balance between the release of eDNA and eRNA and their respective nucleases, which are required to maintain homeostasis. In COVID-19, a significant increase in NETs is observed, and therefore an excess of eDNA. In clinical trials, the human DNase I enzyme, (Dornase Alpha) is being evaluated to reduce the severe symptoms of COVID-19 [56].

### 1.4. Platelets-SARS-CoV-2/Angiotensin-Converting Enzyme 2

Thrombocytopenia in COVID-19 is an indicator of poor prognosis, particularly when it decreases in the first 7 days after admission to the hospital. Thrombocytopenia is an independent risk factor associated with in-hospital mortality. Liu et al. [57] found that an increase of about 50 × 109 /L over the whole range of platelet, decreases mortality. Adding support to the means of platelet activation, several studies show that platelets are activated in COVID-19 patients [58], i.e., there is an increase in young immature platelets named reticulated platelets (RPs). These are associated with high platelet turnover and arterial thrombotic events. In COVID-19, PRs or the immature platelet fraction (IPF) are similar to patients with acute myocardial infarction [59].

Considering that the expression of ACE2 and transmembrane protease serine 2 (TMPRSS2) on human platelets has been detected by immunoblotting, confocal microscopy [60], and flow cytometry [9], a controversial activation route takes into consideration platelet expression of ACE2 and TMPRSS2 receptors. Zhang et al. [9] showed that spike protein binds directly to ACE2, inducing platelet activation and potency in the presence of agonists such as thrombin. However, others such as Manne et al. [61] have detected platelets with mRNA from the SARS-CoV-2 N1 gene in COVID-19, but not the ACE2 receptor. These authors found changes in platelet gene expression and functions associated with ubiquitination, antigen presentation, and mitochondrial dysfunction with increased MAPK pathway activation and thromboxane generation resulting in platelet hyperreactivity [61]. Zaid et al. [62] have also reported that SARS-CoV-2 RNA is associated with platelets similar to other viral infections, however they question the presence of the ACE2 receptor in platelets, due to the methods used to detect these receptors. Moreover, the platelets are the target of infection or capture the viral RNA. On the other hand, Shen et al. [63], using an immunofluorescence assay, flow cytometry analysis, quantitative analysis of SARS-CoV-2 RNA in culture, and Western blot, did not find ACE2 receptors in human platelets and megakaryocytes. In addition, they showed that SARS-CoV-2 could interacts with platelets and megakaryocytes through an ACE2-independent mechanism.

Furthermore, platelets from subjects with severe forms of SARS-CoV-2 infection have increased expression of CD62P and release of thromboxane A2 [58], as well as the formation of platelet-neutrophil, platelet-monocyte, platelet-CD4 T cell, and platelet-CD8 T cell aggregates [61]. Platelets have a procoagulant phenotype [64] generated in the vascular circulation or megakaryocytes, which are affected by SARS-CoV-2 [65].

Platelet hyperactivity in COVID-19 can result from increased protein kinase C phosphorylation, the release of platelet extracellular vesicles, inflammatory cytokines PF4, TNF-α, IL-8, and IL-1β from platelets, inducing the formation of leukocyte-platelet aggregates [62,66].

In addition, platelets also participate with immune complexes or through other mechanisms, as indicated in Table 1 and Table 2.

### 1.5. Heparin-Induced Thrombocytopenia

Considering that one of the main characteristics of COVID-19 is hypercoagulability, and therefore the increased risk of venous and arterial thrombosis, it is necessary to differentiate from Heparin-induced thrombocytopenia (HIT) [67,68,69], particularly secondary to the use of vaccines [70]. Uaprasert et al. [71], in a systematic review and meta-analysis, found a pooled incidence of HIT of 0.8%, being slightly higher in critically ill COVID-19 patients.

HIT is characterized by a decrease in platelets >30–50% associated with thromboembolic complications in around 50% of patients with confirmed HIT. This occurs between 5 and 14 days after starting heparin [72,73].

In the pathogenesis of HIT, antibodies that recognize complexes formed by platelet factor 4 (PF4) and polyanions, such as heparin [74] and anti-protamine (PRT)/heparin, are implicated [75]. These immunogenic complexes induce a response in which IgG bind to platelet Fcγ RIIa receptors. This results in platelet hyperactivity, where they release circulating PF4-bearing microparticles [76], inducing the expression of TF by human monocytes [77] and the release of NETs [78].

HIT has some similarities to the novel disorder, named “vaccine-induced immune thrombotic thrombocytopenia”. For example, it starts 5–20 days after ChAdOx1 nCov-19 vaccination. The difference is that the neoantigen is formed by PF4 with components of the vaccine. Specifically, it is associated with an adenovirus hexon protein/ PF4 complex [79].

## 2. Cytokine Storm Syndrome

During a COVID-19 infection, immune cells flood the lungs and attack them instead of protecting them. The imbalance between PAMPs and pattern recognition receptors (PRRs) could fall into cytokine storm syndrome (CSS) or a specific syndrome from the family of conditions characterized by a cytokine storm such as macrophage activation syndrome. This is associated with autoimmune disorders, hemophagocytic lymphohistiocytosis genetic or secondary to different disorders, and cytokine release syndrome [87]. CSS is also associated with delayed secretion of type I and III interferons [88], and low levels thereof [89], probably due to membrane protein in SARS-CoV-2 via RIG-I/MDA-5-MAVS signaling [90]. Furthermore, CSS has hypercytokinemia [91], macrophage polarization from M2 to M1 [92], with activation of the Plcγ2 pathway and a reduction in Tmem178 levels in macrophages [93], T-cell cytotoxicity defects [16], complement activation [94], and increased NETs [95,96]. As a result of all these changes, patients suffer from hyperinflammation [97], cytokine release [98], cytokine storms [99,100], multiorgan disease [101], and thrombosis [15]. Therefore, various inhibitors have been suggested [102], several of which are being used in clinical trials. Some preliminary studies report a significant reduction in mortality, by Anakinra [103] and Canakinumab [104] for IL-1 inhibition, and baricitinib [105] and ruxolitinib [106] for JAK inhibition. However, others have divergent findings, e.g., tocilizumab [107,108] and sarilumab [109] for IL-6 inhibition (Figure 1). Moreover, molecules with immunomodulatory and antiviral properties that modulate cytokines and interferons in immunosuppressed subjects such as inosine pranobex [110,111] have been used in clinical trials with promising results [112].

## 3. Influence of Heparanase, Heparin and Heparinoids in Complications from COVID-19

Heparanase (HPSE) is an endo-β-D-glucuronidase that has specificity for HS and heparin polysaccharide chains. It participates in the metabolism of HS in the extracellular matrix and its activity is modified in inflammation, cancer and cell migration [113]. Acting as a cofactor of TF, HPSE increases the activity of FX and interacts with the TF pathway inhibitor, acting as a procoagulant [114]. It also has platelet hyperactivity and thrombotic activity [115]. HPSE is increased in COVID-19 patients and is related to pathogenicity [116]. HPSE interacts with other molecules such as RNA, causing vascular leakage and inflammation. Low-molecular-weight heparins (LMWH) are potent inhibitors of HPSE, thrombus, and inflammation [101,117], and they also neutralize histones [118].

Histone levels together with HPSE levels may explain interindividual sensitivities to heparin (unfractionated heparin (UFH) and LMWH) or heparinoids in COVID-19 patients [119,120]. This means that the heparin used to treat microthrombosis in these subjects also participates in the inhibition of histones and could decrease its toxicity. The effect of LMWH is not always sufficient, as mentioned above, or it could be reversed [121]. In COVID-19 patients, the use of LMWH is very important due to its anticoagulant, antiviral, and anti-inflammatory effects [122]. However, the need for higher doses of LMWH has been observed in critically ill patients [123], and the use of oral anticoagulants is required, namely dabigatran, apixaban and rivaroxaban [124,125].

Heparin/ heparan sulphate competes with SARS-CoV-2 and reduces its entry into the body [126,127], because, in the SARSCoV-2 spike (S) protein, the receptor-binding domain (RBD) in the S1 subunit has an ectodomain that interacts with heparan sulfate (HS) [128], and the RBD region in S protein in SARS-CoV-2 interacts with 2-O or 6-O sulphate groups of heparin or enoxaparin [129]. Additionally, it has been reported that heparan sulphate inhibitors, such as mitoxantrone, sunitinib, and BNTX, could block entry of the virus [130]. In general, high negatively charged proteins, such as heparin, protein C, and pentraxin, neutralize histones [131]. Heparin has been shown to act directly against circulating histones, but its action does not depend on its anticoagulant function [132].

Among the heparin derivatives that have been proposed for the treatment of COVID-19 are heparinoids. Sulodexide is a heparinoid containing 80% iduronyl glycosaminoglycan sulphate (IGS) or fast-moving heparin and 20% dermatan sulphate (DS). IGS interacts with and increases antithrombin and heparin cofactor II (HCII) [133], while DS interacts with HCII. This combination has properties resembling those of UFH [134]. Sulodexide also has profibrinolytic activity, which reduces the neo-synthesis of proinflammatory cytokines and inhibits histones [135]. It releases an inhibitor of the endothelial TF pathway, inhibiting FVIIa and FXa [136,137]. The prolonged use of sulodexide produces a “release and depletion” effect as observed with UFH [138]. Nevertheless, the use of heparin also has some limitations, such as its inability to inactivate antithrombin-heparin complex when it is bound to fibrin. Dermatan sulphate-HCII complex has been reported to inactivate fibrin-bound thrombin [139]. The RBD region in the S protein in SARS-CoV-2 interacts with 2-O or 6-O sulphate groups of heparin or enoxaparin [129]. Therefore, heparin, and most likely heparinoids, inhibit cellular interaction with the virus.

It is evident that, in addition to the factors related to the pathogenesis of SARS-CoV-2, other pathogenic factors are associated with thrombosis and inflammation, such as HPSE, eDNA, eRNA, micro-RNAs, and histones. Many of these molecules are being studied in order to find drugs to treat COVID-19.

## 4. Conclusions

During COVID-19 infection, SARS-Cov-2 interacts with ACE2, NRP1, endothelial cells, platelets, NETs, thrombin, eDNA, and histones, inducing heterogeneous clinical manifestations characterized by endothelial damage, microthrombosis, and inflammation. In summary, a network of cells and molecules identified in this axis are simultaneously but differentially affecting COVID-19 patients at different stages of the disease.

## Figures and Tables

**Table 1:** Molecules, cells, complexes, or aggregates of importance in the generation of hypercoagulability and thrombosis.

**Molecules, Cells, Complexes or Aggregates**	**Multiple Cells**		**Neutrophil Extracellular Traps and Histones**			
	Tissue thromboplastin or TF release	Monocyte-platelet and neutrophil-platelet aggregates	Complement- TF–NETs	Remnants of NETs	Neutrophil-platelets	Histones
**Research Type**	Cross-sectional study and brief report using Dual RNA in situ hybridization and immunofluorescence	Comparative study	Clinical trial	Case–control study	Clinical trial	Translational study
**Study Characteristics**	66 patients with COVID-19 and 11 autopsies of lung tissues in patients with COVID-19 associated ARDS	37 patients with SARS-CoV-2 pneumonia and 28 healthy subjects	25 patients hospitalized with COVID-19 and 10 healthy age- and sex-matched individuals served as controls	44 patients hospitalized with COVID-19 who developed thrombosis, and gender- and age-matched COVID-19 patients without clinical thrombosis	36 patients with COVID-19 and 31 healthy controls were studied. Platelet and leukocyte activation, NETs and matrix metalloproteinase 9, a neutrophil-released enzyme, were measured	113 patients with COVID-19
**Key Findings**	Antithrombin/FVIIa complex and TF-containing microparticles were elevated in plasma of patients. TF expression correlated with SARS-CoV-2 staining, also, in regions close to TF, fibrin thrombi and thrombi positive for PF4 in COVID-19 versus non-COVID-19 ARDS lungs was found.	Circulating platelets from subjects with COVID-19 pneumonia show a phenotypic and functional profile of hypercoagulability and promote the activation of factors XII and VIII.	High levels of myeloperoxidase (MPO)/DNA complexes correlated with thrombin-antithrombin (TAT) Activity. Thrombin inhibition (dabigatran) or NETosis inhibition or C5aR1 (C5aRa/PMX-53) blockade decreased platelet-mediated NETs thrombogenicity.	Thrombosis in COVID-19 was associated with higher levels of cell-free DNA, myeloperoxidase-DNA complexes, and citrullinated histone H3 and calprotectin.	Platelet (P-selectin, soluble platelet P-selectin, Circulating CD66b+CD41+ platelet-neutrophil complexes) and neutrophil (neutrophil-derived microparticles, Myeloperoxidase (MPO)–DNA complexes) activation are key features of patients with COVID-19. NETs biomarkers may guide low-molecular-weight heparin treatment.	High levels of circulating histones (>30 μg/mL) in viral infection. Circulating histone levels were significantly higher in non-survivors than those who survived.
**Reference**	[80]	[64]	[18]	[81]	[82]	[83]

*Abbr*: Tissue factor (TF); neutrophil extracellular traps (NETs); acute respiratory distress syndrome (ARDS); platelet factor 4 (PF4).

**Table 2:** Molecules and complexes of importance in the generation of hypercoagulability and thrombosis.

**Molecules and Complexes**	**SARS-CoV-2 Antibodies**			
	SARS-CoV-2 spike/anti-spike IgG immune complexes	Anti-SARS-CoV-2 spike IgG immune complexes dependent on FcγRIIA	Anti-PF4/heparin IgG antibodies	Antiphospholipid antibodies
**Research Type**	In vitro experimental study using recombinant anti-spike IgG, platelet adhesion assay, light transmission aggregometry and flow cytometry.	In vitro experimental study using platelet adhesion assay, in-vitro thrombus formation, light transmission aggregometry, and flow cytometry measurement of fibrinogen binding.	Brief report/case analysis	Cross-sectional cohort study
**Study Characteristics**	SARS-CoV-2 S1 and anti-spike IgG immune complexes with different degrees of glycosylation were evaluated	Effects of low fucosylation and high galactosylation of anti spike IgG immune complex on platelet activation and thrombus formation on vWF were evaluated	12 COVID-19 patients with HIT	Serum samples from 172 hospitalized COVID-19 patients were evaluated for subtypes of aPL antibodies: aCL IgG, IgM, and IgA; anti–β2 glycoprotein I IgG, IgM, and IgA; and aPS/PT IgG and IgM. In addition, IgG purified from COVID-19 patient serum was injected into mouse models.
**Key Findings**	SARS-CoV-2/anti-spike IgG immune complexes increase platelet-mediated thrombosis if IgG expresses both low fucosylation and high galactosylation.	Immune complexes containing afucosylated IgG activate platelet FcγRIIA. Clustering of this platelet FcγRIIA could be inhibited by fostamatinib, ibrutinib or cangrelor that counteracted tyrosine kinases Syk, Btk or P2Y12 respectively.	Increased levels of anti-PF4/heparin antibodies, with negative platelet-activating antibodies.	52% of serum samples have antiphospholipid antibodies IgG fractions purified from serum of patients with COVID-19 could trigger aPL antibody–mediated prothrombotic NETs release and accelerate thrombosis in mouse by increased expression of NET remnants and citrullinated histone H3.
**Reference**	[9]	[84]	[85]	[86]

*Abbr*: Platelet factor 4 (PF4); heparin-induced thrombocytopenia (HIT); Antiphospholipid antibodies (aPL antibodies); anticardiolipin antibodies (aCL); anti-phosphatidylserine/prothrombin (aPS/PT); Platelets and peripheral blood mononuclear cells (PBMCs).
